# Publisher Correction: Ecological control of nitrite in the upper ocean

**DOI:** 10.1038/s41467-019-12252-z

**Published:** 2019-10-10

**Authors:** Emily J. Zakem, Alia Al-Haj, Matthew J. Church, Gert L. van Dijken, Stephanie Dutkiewicz, Sarah Q. Foster, Robinson W. Fulweiler, Matthew M. Mills, Michael J. Follows

**Affiliations:** 1 Department of Earth, Atmospheric and Planetary Sciences, Massachusetts Institute of Technology, Cambridge, MA 02139 USA; 2 Department of Biological Sciences, University of Southern California, Los Angeles, CA 90089 USA; 3 Department of Earth and Environment, Boston University, Boston, MA 02215 USA; 4 Flathead Lake Biological Station, University of Montana, Polson, MT 59860 USA; 5 Department of Earth System Science, Stanford University, Stanford, CA 94305 USA; 6 Department of Biology, Boston University, Boston, MA 02215 USA

**Keywords:** Microbial biooceanography, Biogeochemistry, Ecological modelling, Microbial ecology

Correction to: *Nature Communications* 10.1038/s41467-018-03553-w, published online 23 March 2018.

The original version of this Article contained an error in Fig. 3c, g, in which the data series in red was incorrectly labelled as ‘NH2- oxid. rate’, rather than the correct ‘NO2- oxid. rate’. This has been corrected in both the PDF and HTML versions of the Article.

