# Supplementary material for: Ecological control of nitrite in the upper ocean
Source: Nat Commun. 2018 Mar 23;9:1206. doi: 10.1038/s41467-018-03553-w (PMC5865239; doi:10.1038/s41467-018-03553-w)
Supplement: Supplementary file 1 — Supplementary Information [file 41467_2018_3553_MOESM1_ESM.pdf]

Supplementary Information

Ecological control of nitrite in the upper ocean

Zakem et al.

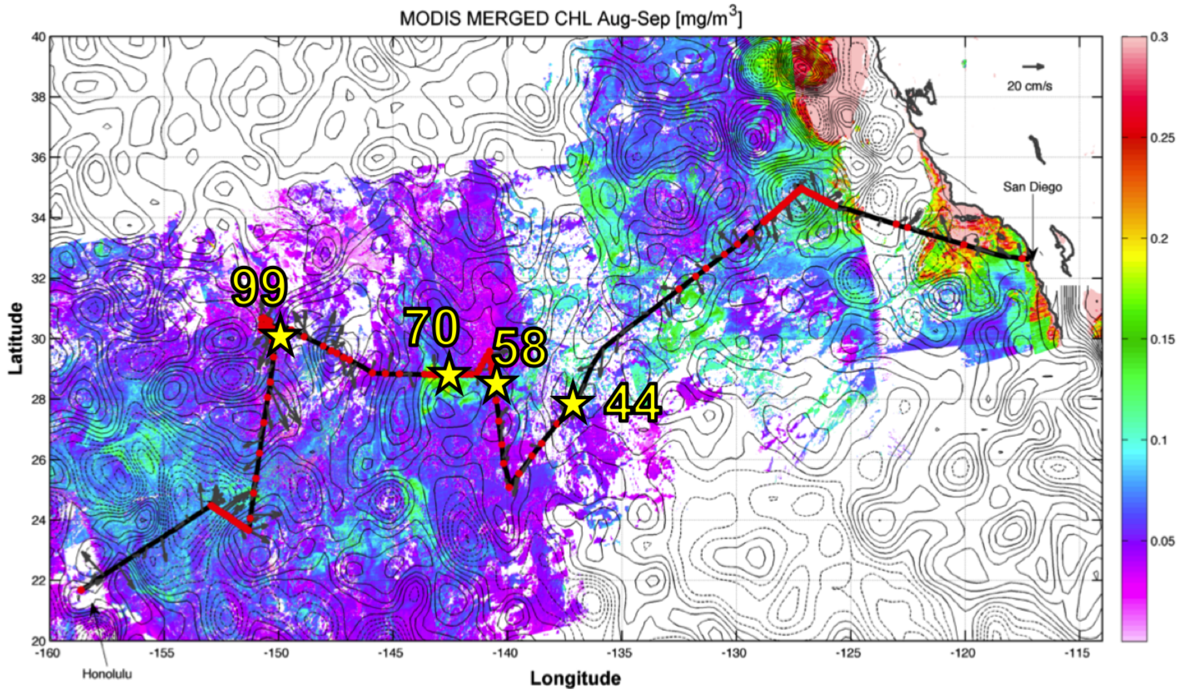

Supplementary Figure 1: Station locations. Locations of the four stations at which nitrification measurements were taken (yellow stars) along the NH1417 cruise track (black line; red dots indicate all stations; Aug.–Sep. 2014). Also shown is surface chlorophyll derived from MODIS-Aqua Level-2[1], representing the week surrounding the ship arrival at each location, and sea surface height contours (Aviso (http://www.aviso.altimetry.fr/): 9/3/14). Grey vectors indicate the horizontal current velocity along the ship track averaged from 50 to 100 m depth and as measured by the 75kHz shipboard ADCP. Chlorophyll and sea surface height data plot courtesy of Daniel Whitt.

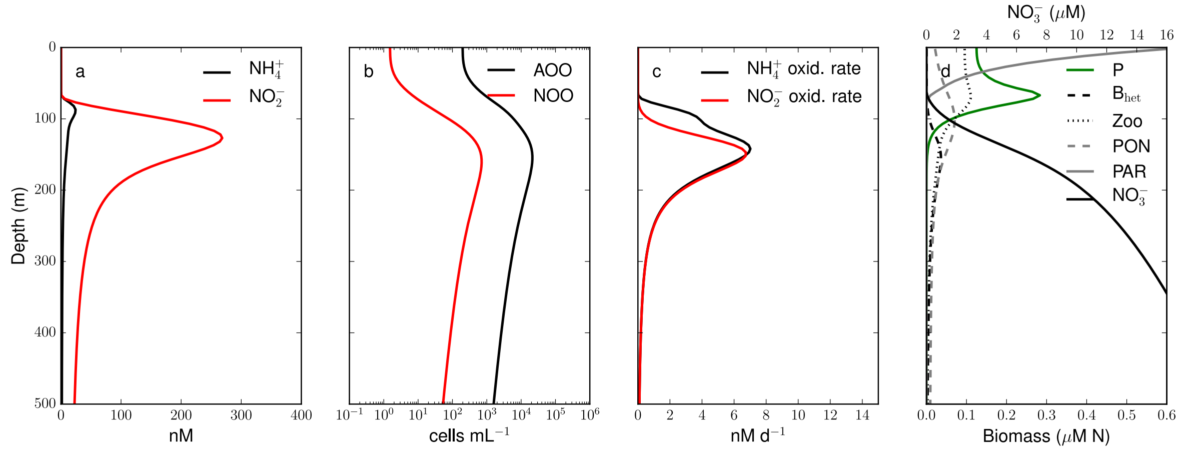

Supplementary Figure 2: Full solutions to the water column model. The solutions illustrated by the solid lines in Fig. 3 in the main text, here showing the entire 2000 m domain of the water column model for all nine state variables. (a) [NH_4^+] and [NO_2^-], (b) ammonia-oxidizing and nitrite-oxidizing organism (AOO and NOO) abundances, (c) nitrification rates, and (d) phytoplankton biomass (P), heterotrophic bacteria biomass (B_{het}), zooplankton biomass (Zoo), particulate organic nitrogen (PON, also in μM), PAR (scaled to fit plot), and [NO_3^-].

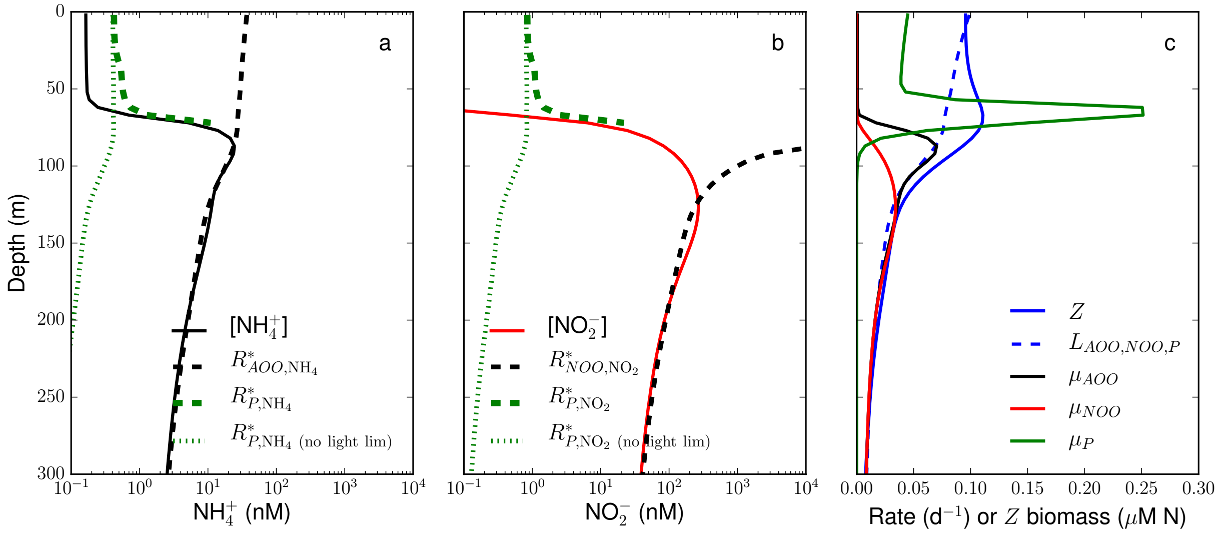

Supplementary Figure 3: Water column model solutions and subsistence concentrations. (a) and (b): As in Fig. 4 in main text, but plotted on log scale, and with the R^*s of P also plotted without the effects of light limitation (i.e., when R^*s are calculated using the constant maximum growth rate μ_{max} instead of μ_{light}, which does not give the increase at the base of the euphotic zone seen in Fig. 4). Since the R^*s of P are so low, the log scale reveals their depth variation from grazing (via L). (c): Microzooplankton grazer biomass Z, loss rates L (equation 29), and resulting steady state growth rates μ. The DIN concentrations are lower in the surface than the computed R^*s because of the effects of vertical mixing. Phytoplankton also continue to sustain a population at the surface even though their growth rate is lower than the loss rate because of vertical mixing. (In reality, lateral supply of nutrients, or another surface process not represented in this idealized model, may sustain higher surface phytoplankton growth rates in oligotrophic regions.) This vertical mixing supplies additional biomass to the surface from the subsurface maxima, driving the actual resource concentration down from the subsistence resource concentration predicted by equations (3) and (28)[2].

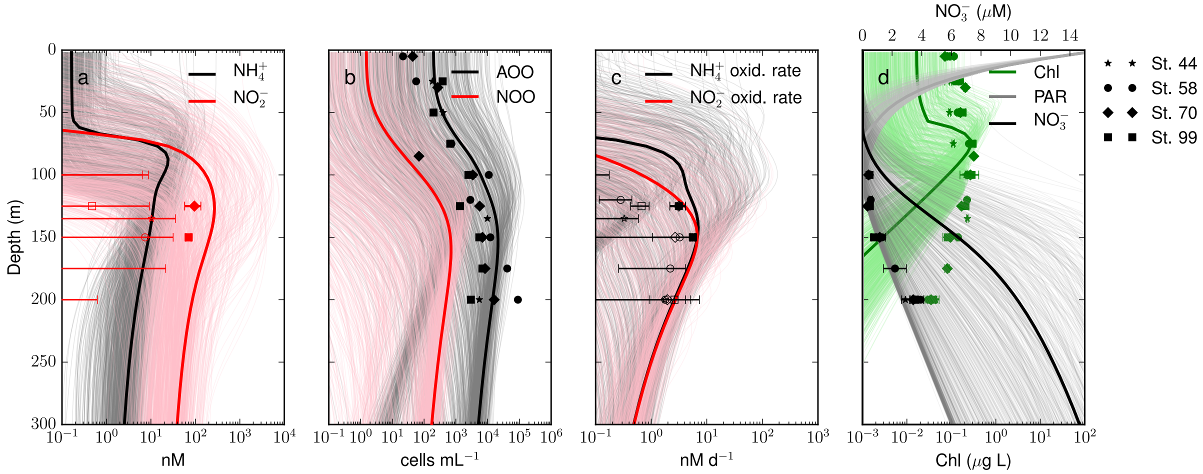

Supplementary Figure 4: Water column model solution ensemble. Thin lines denote each solution from the model ensemble in which parameters of the solution in bold lines were varied according to Table 1. Observations (marked points with error bars in a–d) from four stations in the oligotrophic North Pacific are also shown. (a) [NH_4^+] and [NO_2^-], (b) nitrifier abundances (observed *amoA* gene abundances), (c) nitrification rates, and (d) [Chl *a*], PAR (scaled for plot), and [NO_3^-]. Observations below the detection limit are indicated with open (vs. filled) markers.

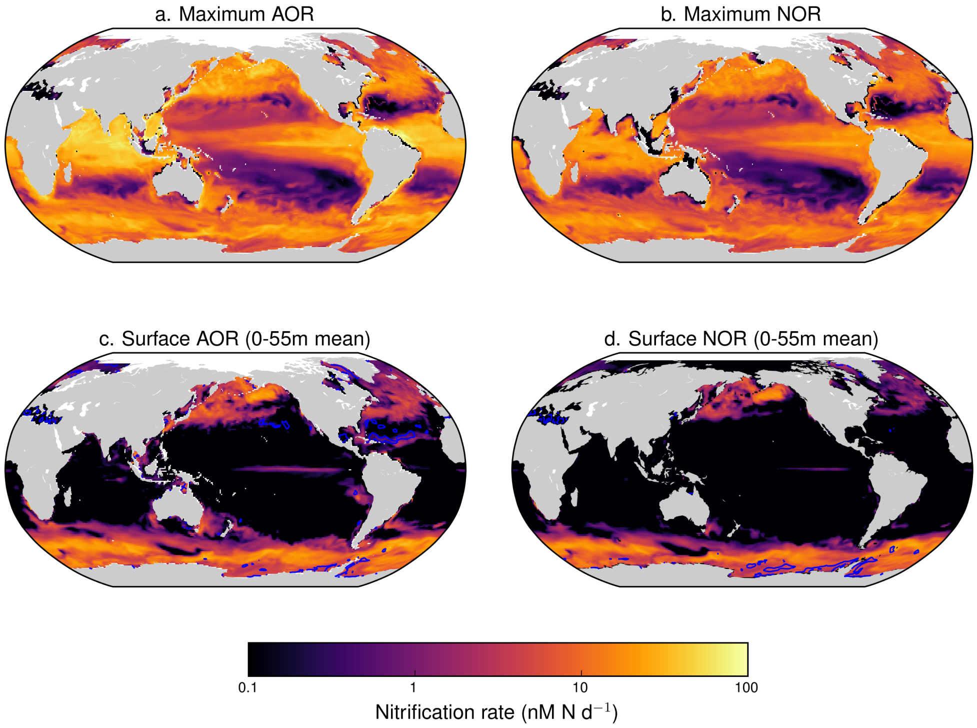

Supplementary Figure 5: Maximum and surface modeled nitrification rates. (a) maximum water column and (b) surface mean NH_4^+ oxidation rates, and (c) maximum water column and (d) surface mean NO_2^- oxidation rates (annually averaged). The blue contour indicates where biological growth rates of the AOO and NOO exactly balance loss rates for the AOR and NOR surface means, respectively, encircling the small areas in which the two metabolisms are 'locally sustainable' (stably co-existing with primary production) on average for the course of one year. This was diagnosed with the net biological rate[3] μ_{NET}.

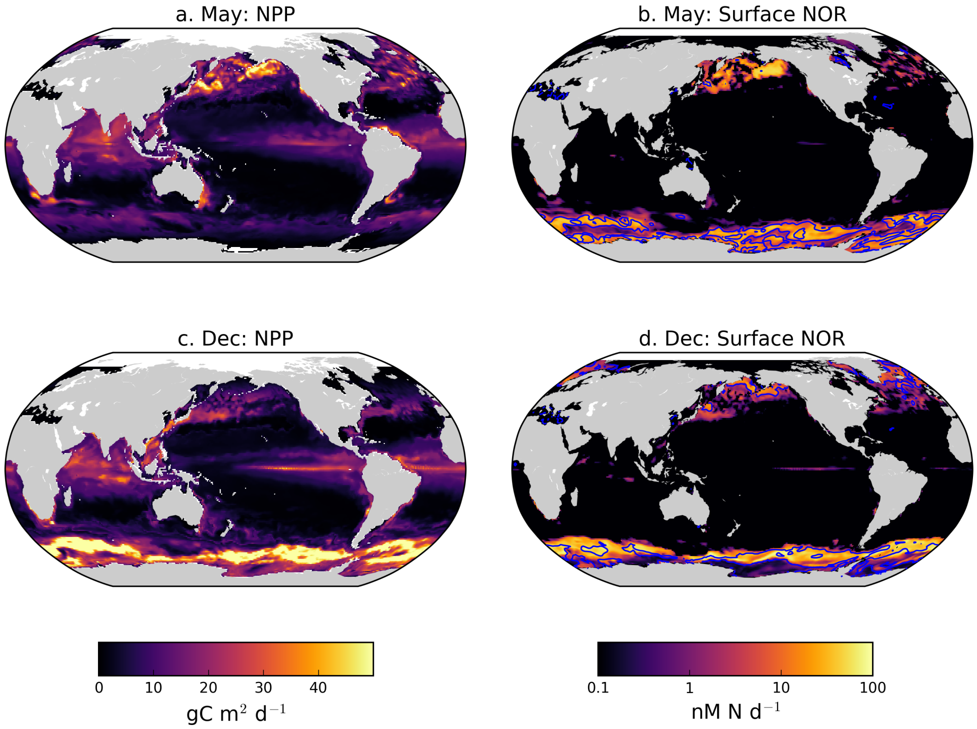

Supplementary Figure 6: Model seasonal resolution of NPP and nitrification. Surface net primary production (NPP) in (a) May and (c) December, and NO_2^- oxidation rate (NOR) in (b) May and (d) December. (Monthly averages are from one model year at the end of the 200 year integration.) The blue contour indicates where biological growth rates of the NOO exactly balance loss rates, encircling the regions where NO_2^- oxidation is a 'locally sustainable' metabolism for that month on average rather than there as a consequence of physical transport[3]. Nitrification rates are higher and more widespread in the early winter in both hemispheres.

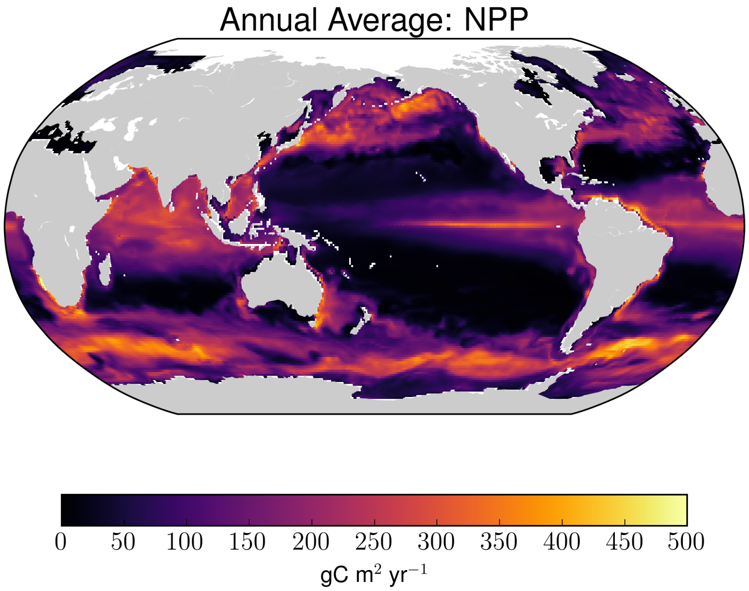

Supplementary Figure 7: Model annual NPP. Annually averaged net primary production (NPP) in the 3D simulation.

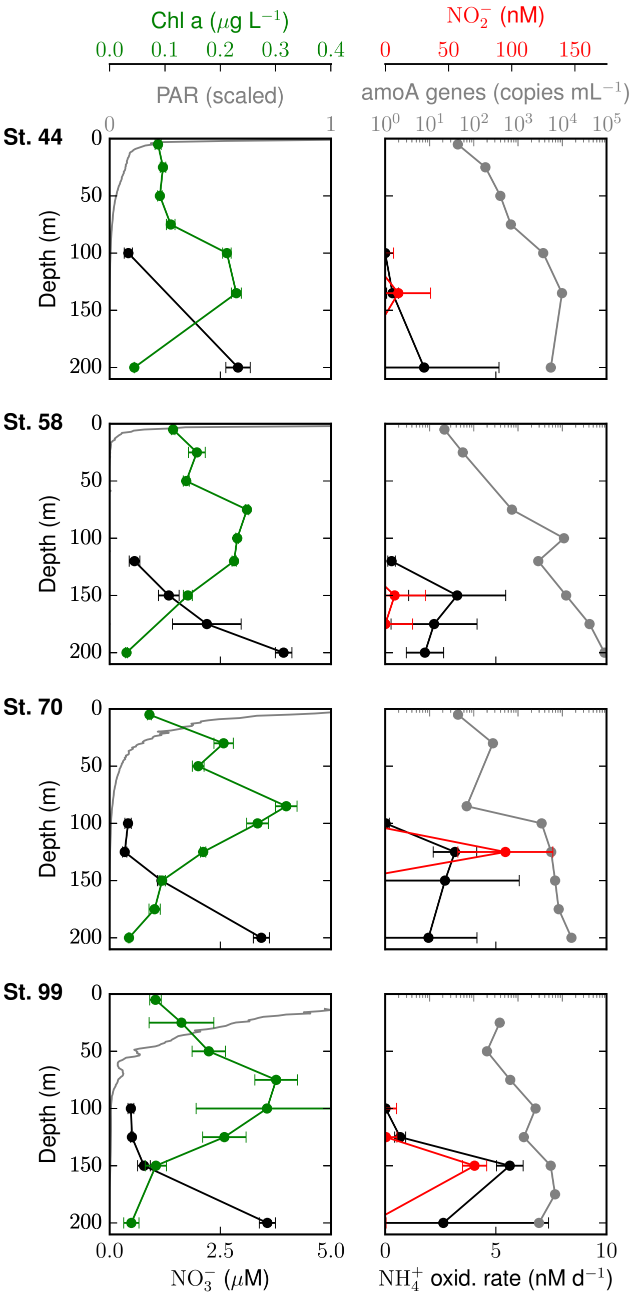

Supplementary Figure 8: Data from the four stations in the oligotrophic North Pacific from cruise NH1417. Chl *a* and NO_3^- concentrations, photosynthetically active radiation (PAR; scaled so that maximum value at each station appears as 1), *amoA* gene abundances, NO_2^- concentrations, and NH_4^+ oxidation rates. For NO_2^- concentrations and NH_4^+ oxidation rates, only two points (125m at Station 70 and 150m at Station 99) were above the detection limit. Error bars denote one s.d.

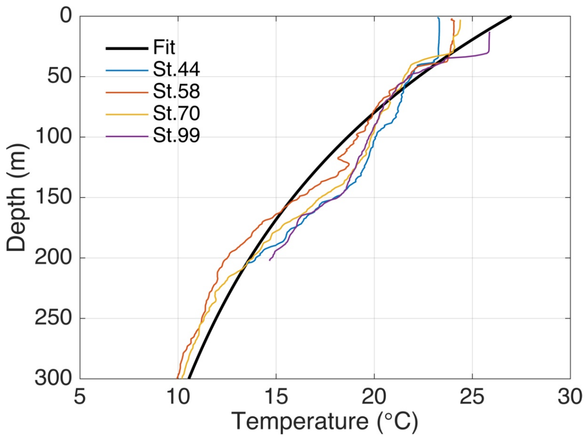

Supplementary Figure 9: Observed and modeled temperature. Observed temperature at the four stations in the North Pacific sampled for nitrification, and the fit used as the temperature field in the water column model.

Supplementary Table 1: Model parameters for 1D and 3D configurations, including the ranges of uncertainty from which values were randomly sampled in the 1D water column model ensemble.

Parameter	Symbol	Value	Range	Units
**Nitrifier growth:**				
NH_4^+ yield, AOO	$y_{\mathrm{NH_4}}$	$(112 \pm 22)^{-1}$	Gaussian	unitless
NO_2^- yield, NOO	$y_{\mathrm{NO_2}}$	$(334 \pm 67)^{-1}$	Gaussian	unitless
Maximum NH_4^+ uptake rate, AOO	$V_{max\mathrm{NH_4},AOO}$	50.8 ± 4.68	Gaussian	mol NH_4^+ mol N^{-1} d^{-1}
Maximum NO_2^- uptake rate, NOO*	$V_{max\mathrm{NO_2},NOO}$	23.6 ± 2.17	Gaussian	mol NO_2^- mol N^{-1} d^{-1}
NH_4^+ half-saturation, AOO	$K_{\mathrm{NH_4},AOO}$	133 ± 38	Gaussian	nM
NO_2^- half-saturation, NOO*	$K_{\mathrm{NO_2},NOO}$	287 ± 82	Gaussian	nM
**Heterotrophic bacterial growth:**				
Maximum N detritus uptake rate, B_{het}	V_{maxD}	1	$\pm 50\%$ Linear (0.5–1.5)	mol N mol N^{-1} d^{-1}
Detritus half-saturation, B_{het}	K_D	0.1	$\pm 50\%$ Linear (0.05–0.15)	μM N
B_{het} yield (detritus)	y_D	0.14	$\pm 50\%$ Linear (0.07–0.21)	unitless
**Phytoplankton growth:**				
Maximum growth rate, P	μ_{max}	0.515	$\pm 50\%$ Linear (0.258–0.773)	d^{-1}
NO_x^- half-saturation, P	K_{NO_xP}	3.6	$\pm 50\%$ Linear (1.8–5.4)	nM
NH_4^+ half-saturation, P	$K_{\mathrm{NH_4}P}$	1.8 ($= 0.5K_{\mathrm{NO_x}P}$)	$= 0.5K_{\mathrm{NO_x}P}$ (0.6–2.7)	nM
Maximum quantum yield	ϕ	0.04		mol C mol^{-1} photons
Chl *a* absorption by P	$a_{\mathrm{phy}}^{\mathrm{chl}}$	0.02		m^2 $(mgChl)^{-1}$
Chl:C maximum	θ_{max}	0.2		g Chl g^{-1} C
**Grazing and mortality:**				
Maximum grazing rate	g_{max}	1	$\pm 50\%$ Linear (0.5–1.5)	d^{-1}
Grazing half-saturation	K_g	1	$\pm 50\%$ Linear (0.5–2)	μM N
Grazing efficiency	ζ	0.5	$\pm 50\%$ Linear (0.25–0.75)	unitless
Mortality rate (AOO/NOO/B_{het}/P)	m_B	$0.01\gamma_T$	$\pm 50\%$ Linear ((0.005–0.015)γ_T)	d^{-1}
Quadratic mortality rate (Z)	m_Z	$0.7\gamma_T$	$\pm 50\%$ Linear ((0.35–1.05)γ_T)	μM N^{-1} d^{-1}
**Temperature dependence:**				
Reference temperature	T_0	293.15		K
Temperature regulation	A_E	-4000		K
Temperature normalization	τ	0.8		unitless
**Physical parameters for 1D model:**				
Maximum incoming PAR flux	I_{max}	1400		W m^{-2}
PAR attenuation in water	k_w	0.04		m^{-1}
PAR attenuation due to chlorophyll (and CDOM)	k_{Chl}	0.04		m^2 $(mgChl)^{-1}$
Mixed-layer attenuation depth	z_{ML}	20		m
Minimum vertical mixing coefficient	K_{min}	$5 \cdot 10^{-5}$		m^2 s^{-1}
Maximum vertical mixing coefficient	K_{max}	10^{-2}		m^2 s^{-1}
Detrital sinking rate (also in 3D)	w_s	10		m d^{-1}

*These values are those used in the "Both yield and affinity differences" default simulation in the 1D water column model (solid red lines in Fig. 3). The yield and/or affinity differences were removed for the other model experiments (dotted and dashed red lines) by using the listed values of the AOO for the NOO. In the illustrated 3D global simulation, the affinity of the NOO was adjusted by a factor of $2^{1/3}$ instead of $10^{1/3}$ as described in the Methods, giving values of $V_{max\mathrm{NO_2},NOO} = 40.3$ d^{-1} and $K_{\mathrm{NO_2},NOO} = 168$ nM.

Supplementary Table 2: Estimated biomass yields with respect to NH_4^+ and NO_2^- from cultures, with electron fraction f calculated from equations (6) and (7).

	Organism	Δ[DIN] (μM)	Cell yield $\left(\frac{10^6\text{cells}}{\text{mL}}\right)$	Cell yield $\left(\frac{10^6\text{cells}}{\mu\text{M DIN}}\right)$	Quota $\left(\frac{\text{fmol N}}{\text{cell}}\right)$	NO_2^- oxid: CO_2 fixed	C:N	y^{-1} $\left(\frac{\text{mol DIN}}{\text{mol N}}\right)$	f
AOO:									
Martens-Habbena et al. (2009)[4]	SCM1	850	44	52	0.12			161	
Qin et al. (2014)[5]	SCM1			112.6	0.12			74	
	HCA1			80.8	0.12			103	
	PS0			70.4	0.12			118	
Santoro and Casciotti (2011)[6]	CN75	40	3.2	80	0.12			104	
**Average y^{-1}**								**112±32**	**0.030 (0.02–0.04)**
Lowest (Q=0.16 fmol N cell^{-1})								56	0.06
Highest (Q=0.07 fmol N cell^{-1})								300	0.01
NOO:									
Spieck et al. (2014)[7]	*N. watsonii*			6.6	1.2			126	
	N. gracilis			7.2	1.2			116	
Watson and Waterbury (1971)[8]	*N. gracilis*					160	5	780	
	N. mobilis					44	5	220	
**Average y^{-1}**								**310±320**	**0.032 (0.02–1)**
Lowest (Q=1.6 fmol N cell^{-1})								87	0.1
Highest (C:N=6)								1080	0.01

Supplementary Note 1: Temperature sensitivity

In the illustrated simulations, temperature does not modify V_{max}, the maximum uptake rate of DIN by AOO and NOO that sets the maximum nitrification rate, in line with experimental evidence[9]. Including a temperature modification changes solutions quantitatively but not qualitatively, with the exception of the oxygenated equatorial regions in the Indian and Atlantic basins in the global simulation. There, the solutions do exhibit a significant temperature sensitivity: if nitrifier rates are allowed to increase with temperature in the same way as the heterotrophic bacteria, the effect of the high modeled NO_2^- concentrations in the Equatorial Atlantic and Indian Oceans (illustrated in Fig. 5) is removed, and the resulting NO_2^- concentrations are more similar to those of the modeled equatorial Pacific as well as to the observations. This may suggest that the observed lack of temperature sensitivity[9] may not apply to all nitrifiers. The temperature dependencies in general have a small but non-negligible effect on ecosystem structure, slowing all microbial rates with depth and with latitude.

Supplementary Note 2: How does vertical mixing affect the magnitude of the PNM?

Though R^* concentrations explain why NO_2^- accumulates, NO_2^- concentrations are higher than R^* in the model. This indicates that transport processes are likely non-negligible in setting the magnitude of the PNM. How can we understand this peak concentration?

The influence of physical transport on R^* can be accounted for by including the advective and diffusive fluxes in the equation for NOO biomass (equation 14). This modified subsistence concentration, $\bar{R}^*$, is:

$$\bar{R}^*{}_{\mathrm{NO_2}NOO} = \frac{K_{\mathrm{NO_2}}(L - \frac{1}{B_{NOO}} J_{B_{NOO}})}{y_{\mathrm{NO_2}} V_{max_{\mathrm{NO_2}}} - (L - \frac{1}{B_{NOO}} J_{B_{NOO}})} \tag{S1}$$

where $J_{B_{NOO}}$ represents both advection and diffusion of NOO biomass ($J_{B_{NOO}} = -\nabla \cdot (\mathbf{u} B_{NOO}) + \nabla \cdot (\mathbf{K} \nabla B_{NOO})$, though in the water column, only a diffusive flux (vertical mixing) affects biomass. Conceptually, this balance reveals that a gradient in NOO biomass at depth (as nitrification becomes

energetically favorable) fuels a diffusive flux of cells away from the PNM, increasing the subsistence concentration from R^* to $\bar{R}^*$. At the PNM, the NOO cannot sustain a population large enough to draw down NO_2^- to the original R^*.

An alternative balance for NO_2^- can be derived from the fluxes of NO_3^- (equation 11). For a steady-state environment, defined as such when the change in total NO_3^- concentration is small relative to its fluxes, three NO_3^- fluxes are in balance: production by NOO, assimilation by phytoplankton ($V_{NO_3}P$), and physical transport. Substituting in equation (2) for NO_2^--limited growth of NOO gives:

$$0 = V_{max_{NO_2}} \frac{\mathrm{NO_2^-}}{\mathrm{NO_2^-} + K_{NO_2}} B_{NOO} - V_{NO_3}P + J_{\mathrm{NO_3}} \tag{S2}$$

where $J_{\mathrm{NO_3}}$ represents both advection and diffusion of NO_3^- ($J_{\mathrm{NO_3}} = -\nabla \cdot (\mathbf{u}\mathrm{NO_3^-}) + \nabla \cdot (\mathbf{K}\nabla\mathrm{NO_3^-})$). Rearranging gives:

$$[\mathrm{NO_2}]_{SS} = \frac{K_{NO_2}(V_{NO_3}P - J_{\mathrm{NO_3}})}{V_{max_{NO_2}} B_{NOO} - (V_{NO_3}P - J_{\mathrm{NO_3}})} \tag{S3}$$

Like $\bar{R}^*$, it does not neglect any terms, and so exactly predicts the NO_2^- concentration throughout the model water column. Like $\bar{R}^*$, reduced NOO affinity results in a higher NO_2^- concentration. Unlike $\bar{R}^*$, the yield is reflected implicitly: the lower yield results in a higher NO_2^- concentration via the lower NOO biomass.

In addition to nitrifier activity, this balance emphasizes that phytoplankton activity and circulation are crucial to setting the PNM. In the surface ocean, if phytoplankton assimilate nearly all of the physically supplied NO_3^-, the difference in $V_{NO_3}P$ and $J_{\mathrm{NO_3}}$ is small, and NO_2^- will approximate zero concentration. This characterizes the surface in the water column model. At the PNM, if phytoplankton are unable to assimilate all of the supplied NO_3^- because they are light-limited, NO_3^- may be transported away from that location ($J_{\mathrm{NO_3}} < 0$). This can be understood as a consequence of the steep gradient in NO_3^- at the onset of the nitricline. This gradient fuels the upward diffusive flux of NO_3^-. There, NO_2^- accumulates, enhancing the PNM beyond R^*.

Notably, phytoplankton excretion of NO_2^-, the alternative hypothesis for the formation of the PNM not considered extensively in this study, would not enter into equation (S3) directly. Rather, this excretion should affect the balance via reduced phytoplankton biomass. Thus equation (S3) holds for all sources and sinks of NO_2^-, including NO_3^- reduction, and so may serve as a useful expression with which to integrate the effects of nitrifiers, phytoplankton, and transport in diverse environments.

Supplementary Note 3: Iron limitation to nitrifier growth

Model experiments that include an iron limitation to just NOO growth, or to both AOO and NOO growth, show very similar solutions to those illustrated in the main text. These preliminary experiments assumed a similar iron affinity and an iron to carbon ratio of biomass for the nitrifiers and picoplankton. Though a full analysis of the iron limitation to nitrifier growth is beyond the scope of this study, one hypothesis is that small size may allow the nitrifiers a higher affinity for iron than the larger phytoplankton that often populate subpolar waters. Also, evidence that AOO require copper rather than iron for redox machinery makes it plausible that at least NH_4^+ oxidation should be favorable in HNLC regions[10]. Thus, we speculate that it may be possible for nitrifiers to thrive in an environment with low iron concentrations despite iron limitation to much of primary production. Future work could examine the hypothesis that the ratio of primary production to nitrification may reflect the relative supply rates of iron and DIN in HNLC regions when light does not limit primary production.

Supplementary Note 4: Daily resolution

If phytoplankton activity is light-limited at night, does nitrification occur in the surface? Supplementary Fig. 10 shows the water column model results over time, with resolution of a daily light cycle. Primary production varies with time of day. At the base of the euphotic zone, both NH_4^+ oxidation and NO_2^- oxidation occur continuously in the model because the timescales of sinking and remineralization of organic matter are sufficiently long as to allow for continual remineralization at depth. Though the model resolves the daily cycle simplistically, it suggests that nitrification does not occur at significantly higher rates at night at the surface.

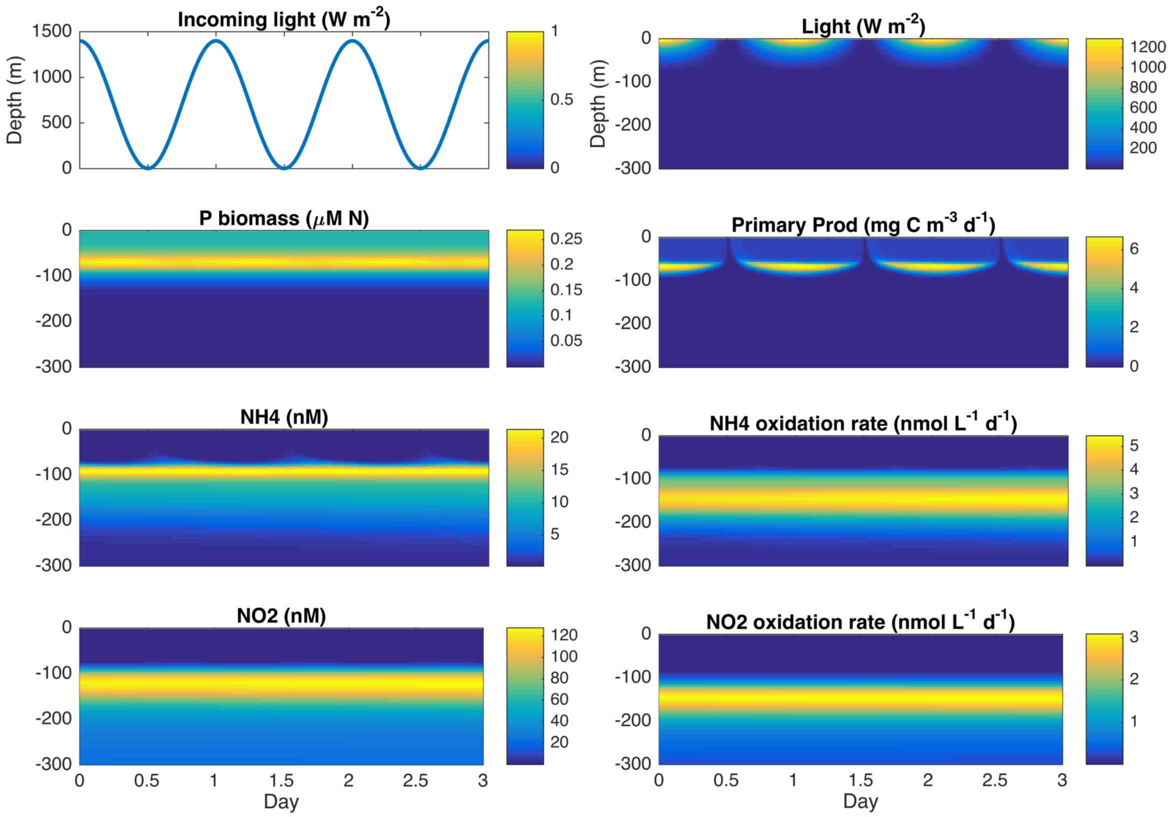

Supplementary Figure 10: Water column model solutions with daily resolution. Solutions plotted as a function of time, showing the daily light cycle.

Supplementary Note 5: More detail on the estimated yields

AOA observations: From Martens-Habbena *et al.* [4], we estimated ΔDIN as the initial and end NH_4^+ concentrations and cell yield from their Fig. 1 for *Nitrosopumilus maritimus* strain SCMI. From Qin *et al.* [5], we used the reported ranges in cell yield per mole of NH_4^+ oxidized (pg. 12507) for growth in organic carbon supplemented media for each of three strains of *Nitrosopumilus maritimus*. From Santoro & Casciotti [6], we estimated the initial and end NH_4^+ concentrations and cell yield from their Fig. 3 for AOA strain CN75.

NOB observations: From Spieck *et al.* [7], we used the reported cell yields (pg. 172) for chemolithoautotrophic growth of the new bacterium, named *Nitrospina watsonii*, and for mixotrophic growth of *Nitrospina gracilis*. From Watson & Waterbury [8], the ratio of NO_2^- oxidized to CO_2 fixed was calculated for the results of each of the two reaction mixtures listed in their Table 2 for *Nitrospina gracilis* and *Nitrococcus mobilis*, the average taken for the reaction mixtures, and a C:N of 5±1 assumed for the conversion to the growth yield[11].

Note on mixotrophy: Mixotrophic growth (i.e., the consumption of organic molecules in addition to DIN) is a known characteristic of both AOO and NOO. Obligate mixotrophy of two strains of AOA (HCA1 and PS0), and facultative mixotrophy of SCMI has been demonstrated[5]. Mixotrophic growth enhanced the yield by 20%. Mixotrophic growth of NO_2^--oxidizer *N. gracillis* was observed to be about 10% higher than chemoautotrophic growth[7]. These results suggest two things: 1. that mixotrophy is common among marine nitrifying microorganisms, and 2. that yields of mixotrophic growth are enhanced, but are of the same order of magnitude as pure chemoautotrophic growth. The latter supports the assumption here that the theoretically-derived yields should hold across variations in metabolism, such as different forms of mixotrophy.

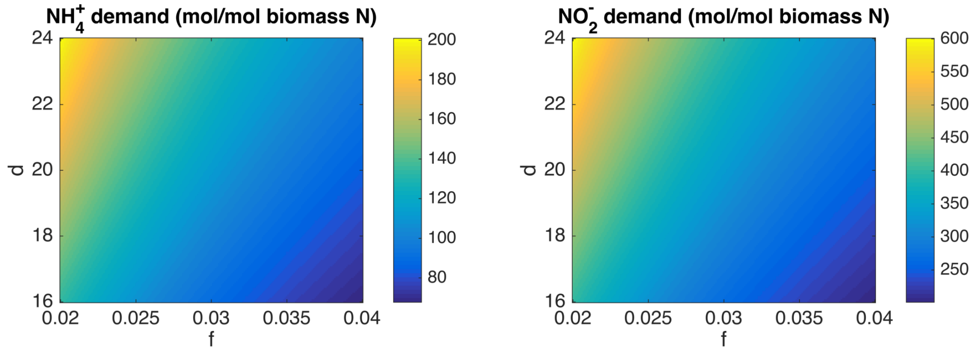

Supplementary Figure 11: NH_4^+ and NO_2^- demand. Prediction of demand (as the inverse of the yield y) for the AOO and NOO metabolic functional types, respectively, as a function of parameters f and d.

Supplementary Note 6: Sensitivity of yields to parameters f and d

In natural assemblages of organisms, we expect wide variation in the approximated stoichiometries in the main text (equations 4 and 5) due to variation in efficiency, biomass composition, energies of synthesis, and versatility of metabolism, but hypothesize that the broad pattern, a significantly higher demand for NO_2^- than NH_4^+ for the same amount of growth, remains.

Supplementary Fig. 11 illustrates the DIN demand, the inverse of the yield y, as a function of f and d for the AOO and the NOO metabolic types, following the full equations for the two metabolisms in the main text (equations 4 and 5). Values correspond to a plausible range in f as guided by the observations, and the range in d for the C:N variation of one given in Zimmerman *et al.* [11]. The midpoint of both ranges is that used in the illustrated model solutions ($f = 0.03, d = 20$).

This plot supports our claim that there should be a broad pattern of significantly higher demand for NO_2^- than NH_4^+ for the same rate of growth of the AOO and NOO metabolic types. The DIN demand is highest (yield y is lowest) for low f and high d. Over the plotted range, the highest NH_4^+ demand for the AOO – about 200 moles NH_4^+ per mole biomass synthesized – is lower than the lowest NO_2^- demand.

Supplementary Note 7: Detail for additional functional types in global model

The framework for the heterotrophic functional type is here described in detail to allow for comparison with other types. Organic matter (OM) provides the elements and electrons for both the synthesis of biomass (B) and energy production, and oxygen serves as the electron acceptor.

$$\frac{1}{d_{OM}}\mathrm{C}_{c_{OM}}\mathrm{H}_{h_{OM}}\mathrm{O}_{o_{OM}}\mathrm{N}_{n_{OM}} + \frac{1-f}{4}\mathrm{O}_2 \rightarrow \frac{f}{d_B}\mathrm{C}_{c_B}\mathrm{H}_{h_B}\mathrm{O}_{o_B}\mathrm{N}_{n_B} + \left(\frac{c_{OM}}{d_{OM}} - \frac{c_B f}{d_B}\right)\mathrm{CO}_2 + \left(\frac{n_{OM}}{d_{OM}} - \frac{n_B f}{d_B}\right)\mathrm{NH}_4^+$$

where d is the number of electron equivalents for the generic organic composition $\mathrm{C}_c\mathrm{H}_h\mathrm{O}_o\mathrm{N}_n$ that correspond to the oxidation states of its inorganic constituents (below as $d = 4c + h - 2o - 3n$). The growth efficiency (mol B mol $^{-1}$ OM, or, mol C synthesized mol $^{-1}$ C consumed) relates to f as:

$$y_{OM} = \frac{d_{OM}}{d_B} f \tag{S4}$$

and so $y_{OM} = f$ when assuming the same stoichiometry for both the organic matter substrate and microbial biomass. When assuming the average stoichiometry of marine organic matter ($\mathrm{C}_{106}\mathrm{H}_{175}\mathrm{O}_{42}\mathrm{N}_{16}$)[12] for both the organic matter substrate and microbial biomass, the full metabolism is represented as:

$$\mathrm{C}_{106}\mathrm{H}_{175}\mathrm{O}_{42}\mathrm{N}_{16} + \frac{467(1-y_{OM})}{4}\mathrm{O}_2 \rightarrow y_{OM}\mathrm{C}_{106}\mathrm{H}_{175}\mathrm{O}_{42}\mathrm{N}_{16} + 106(1-y_{OM})\mathrm{CO}_2 + 16(1-y_{OM})\mathrm{NH}_4^+$$

The stoichiometries for the three anaerobic metabolic functional types are as follows:

Nitrate-reducing heterotrophy For the NO_3^- reducer, organic matter (OM) provides the elements and electrons for both the synthesis of biomass (B) and energy production, and NO_3^- serves as the electron acceptor, which is reduced to NO_2^-. The full metabolism forming NO_3^--reducing biomass B_{HetNO_3}, here written in terms of OM and B_{HetNO_3} for brevity, is:

$$
\begin{aligned}
&\frac{1}{d_{OM}} OM + \frac{1-f}{2} \mathrm{NO_3^-} \\
&\qquad\to \quad \frac{f}{d_B} B_{HetNO_3} + \left(\frac{c_{OM}}{d_{OM}} - \frac{c_B f}{d_B}\right) \mathrm{CO_2} \\
&\qquad\qquad + \left(\frac{n_{OM}}{d_{OM}} - \frac{n_B f}{d_B}\right) \mathrm{NH_4^+} + \frac{1-f}{2} \mathrm{NO_2^-}
\end{aligned}
$$

For marine stoichiometry, the full metabolism is:

$$
\begin{aligned}
&\mathrm{C_{106}H_{175}O_{42}N_{16}} + \frac{467(1-y_{OM})}{2} \mathrm{NO_3^-} \\
&\qquad\to \quad y_{OM}\mathrm{C_{106}H_{175}O_{42}N_{16}} + 106(1-y_{OM})\mathrm{CO_2} + 16(1-y_{OM})\mathrm{NH_4^+} \\
&\qquad\qquad + \frac{467(1-y_{OM})}{2} \mathrm{NO_2^-}
\end{aligned}
$$

Denitrifying heterotrophy For the denitrifier, organic matter (OM) provides the elements and electrons for both the synthesis of biomass (B) and energy production, and NO_2^- serves as the electron acceptor, which we consider here as all being reduced completely to N_2 (here neglecting the formation of N_2O). The full metabolism forming denitrifying biomass B_{HetNO_2} is:

$$
\begin{aligned}
&\frac{1}{d_{OM}} OM + \frac{1-f}{3} \mathrm{NO_2^-} \\
&\qquad\to \quad \frac{f}{d_B} B_{HetNO_2} + \left(\frac{c_{OM}}{d_{OM}} - \frac{c_B f}{d_B}\right) \mathrm{CO_2} \\
&\qquad\qquad + \left(\frac{n_{OM}}{d_{OM}} - \frac{n_B f}{d_B}\right) \mathrm{NH_4^+} + \frac{1-f}{6} \mathrm{N_2}
\end{aligned}
$$

For marine stoichiometry, the full metabolism is:

$$\begin{aligned} C_{106}H_{175}O_{42}N_{16} + \frac{467(1-y_{OM})}{3}NO_2^- \\ \rightarrow \quad & y_{OM}C_{106}H_{175}O_{42}N_{16} + 106(1-y_{OM})CO_2 + 16(1-y_{OM})NH_4^+ \\ & + \frac{467(1-y_{OM})}{6}N_2 \end{aligned}$$

The organic matter yield for these two anaerobic heterotrophic types is assigned at 0.13 (mol biomass synthesized mol organic matter^{-1}). The fact that this yield is lower than that of the aerobic heterotrophic type results in the competitive exclusion of both anaerobic heterotrophs in oxygenated environments (above about 10 nanomolar). The difference in stoichiometry for the DIN demand and excretion between the NO_3^--reducer and denitrifier results in some accumulation of NO_2^- in the water column where both steps of anaerobic heterotrophy occur.

Anammox For chemoautotrophic anaerobic ammonium oxidation (anammox), NH_4^+ oxidation to elemental N provides electrons for energy that fuels cell synthesis (here considering NH_4^+ and NH_3 interchangeably), and NO_2^- serves as the electron acceptor. Together, elemental N_2 is formed. The three half-reactions are:

$$\begin{aligned} (1) \quad & \left[\frac{1}{3}NH_4^+ \rightarrow \frac{1}{6}N_2 + \frac{4}{3}H^+ + e^-\right] \\ (1-f) \quad & \left[\frac{1}{3}NO_2^- + \frac{4}{3}H^+ + e^- \rightarrow \frac{1}{6}N_2 + \frac{2}{3}H_2O\right] \\ (f) \quad & \left[\frac{n}{d}NH_4^+ + \frac{c-n}{d}CO_2 + \frac{n}{d}HCO_3^- + H^+ + e^- \right. \\ & \left. \rightarrow \frac{1}{d}C_cH_hO_oN_n + \frac{2c-o+n}{d}H_2O\right] \end{aligned}$$

which gives the full metabolism forming anammox biomass B_{anmx} when summed as:

$$\left(\frac{1}{3}+\frac{f}{d}\right)NH_4^+ + \frac{1-f}{3}NO_2^- + \frac{cf}{d}CO_2 \rightarrow \frac{f}{d}B_{anmx} + \frac{2-f}{6}N_2$$

A value of f of 0.05, higher than that of the nitrifiers, results in very similar stoichiometry to that measured and reported by Strous *et al.* [13], but perhaps better reflects optimal laboratory conditions rather than the mesopelagic ocean. However, assuming either $f = 0.03$ (the same value for the nitrifiers) or $f = 0.05$ results in the exclusion of anammox bacteria from the oxygenated ocean. With $f = 0.03$, the anammox metabolism, normalized to one mole of N in order to compare with the nitrifier metabolisms, is:

$$223\mathrm{NH}_4^+ + 216\mathrm{NO}_2^- + 5\mathrm{CO}_2 \rightarrow B_{anmx} + 219\mathrm{N}_2$$

Supplementary References

1. NASA Goddard Space Flight Center, Ocean Ecology Laboratory, Ocean Biology Processing Group. Moderate-resolution Imaging Spectroradiometer (MODIS) Aqua Ocean Color Data; 2014 Reprocessing. NASA OB.DAAC, Greenbelt, MD, USA, doi: 10.5067/AQUA/MODIS_OC.2014.0.
2. Lévy, M., Jahn, O., Dutkiewicz, S. & Follows, M. J. Phytoplankton diversity and community structure affected by oceanic dispersal and mesoscale turbulence. *Limnol. Oceanogr. Fluids Environ.* **4,** 67–84 (2014).
3. Clayton, S., Dutkiewicz, S., Jahn, O. & Follows, M. J. Dispersal, eddies, and the diversity of marine phytoplankton. *Limnol. Oceanogr. Fluids Environ.* **3,** 182–197 (2013).
4. Martens-Habbena, W., Berube, P. M., Urakawa, H., de la Torre, J. R. & Stahl, D. A. Ammonia oxidation kinetics determine niche separation of nitrifying Archaea and Bacteria. *Nature* **461,** 976–9 (2009).
5. Qin, W. *et al.* Marine ammonia-oxidizing archaeal isolates display obligate mixotrophy and wide ecotypic variation. *Proc. Natl. Acad. Sci.* **111,** 12504–12509 (2014).
6. Santoro, A. E. & Casciotti, K. L. Enrichment and characterization of ammonia-oxidizing archaea from the open ocean: phylogeny, physiology and stable isotope fractionation. *ISME J.* **5,** 1796–808 (2011).
7. Spieck, E., Keuter, S., Wenzel, T., Bock, E. & Ludwig, W. Characterization of a new marine nitrite oxidizing bacterium, Nitrospina watsonii sp. nov., a member of the newly proposed phylum "Nitrospinae". *Syst. Appl. Microbiol.* **37,** 170–176 (2014).
8. Watson, S. W. & Waterbury, J. B. Characteristics of two marine nitrite oxidizing bacteria, Nitrospina gracilis nov. gen. nov. sp. and Nitrococcus mobilis nov. gen. nov. sp. *Arch. Mikrobiol.* **77,** 203–230 (1971).
9. Horak, R. E. a. *et al.* Ammonia oxidation kinetics and temperature sensitivity of a natural marine community dominated by Archaea. *ISME J.* **7,** 2023–33 (2013).
10. Stahl, D. A. & de la Torre, J. R. Physiology and diversity of ammonia-oxidizing archaea. *Annu Rev Microbiol* **66,** 83–101 (2012).
11. Zimmerman, A. E., Allison, S. D. & Martiny, A. C. Phylogenetic constraints on elemental stoichiometry and resource allocation in heterotrophic marine bacteria. *Environ. Microbiol.* **16,** 1398–1410 (2014).
12. Anderson, L. A. On the hydrogen and oxygen content of marine phytoplankton. *Deep Sea Res. Part I Oceanogr. Res. Pap.* **42,** 1675–1680 (1995).
13. Strous, M., Heijnen, J. J., Kuenen, J. G. & Jetten, M. S. M. The sequencing batch reactor as a powerful tool for the study of slowly growing anaerobic ammonium-oxidizing microorganisms. *Appl. Microbiol. Biotechnol.* **50,** 589–596 (1998).
