# Supplementary material for: Ecological control of nitrite in the upper ocean
Source: Nat Commun. 2018 Mar 23;9:1206. doi: 10.1038/s41467-018-03553-w (PMC5865239; doi:10.1038/s41467-018-03553-w)
Supplement: Supplementary file 3 — Description of Additional Supplementary Files [file 41467_2018_3553_MOESM3_ESM.pdf]

Description of Supplementary Files

File Name: Supplementary Data 1
Description: The observations presented in the main article (Fig. 3) during Cruise NH1417 in August and September 2014: NH_4^+ oxidation rates, [NO_2^-], [NO_3^-], *amoA* gene abundances, and Chl *a* concentrations from four stations in the subtropical North Pacific.
